# Dietary diversity among women of reproductive age (15–49 yrs) across rural and urban settings: a scoping review

**DOI:** 10.3389/fnut.2026.1893369

**Published:** 2026-10-08

**Authors:** Sujata Murarkar, Jayashree Gothankar

**Affiliations:** Department of Community Medicine, Bharati Vidyapeeth (DTU) Medical College, Pune, India

**Keywords:** dietary diversity, micronutrient deficiencies, reproductive age, rural area, urban informal settlement, women

## Abstract

Dietary diversity (DD) reflects micronutrient adequacy and diet quality. Inadequate dietary diversity has adverse effects on health of the women and increases the risk of complications during pregnancy and childbirth due to deficiencies of micronutrients. This scoping review aims to synthesize the current understanding of dietary diversity among WRA in urban and rural settings, highlighting key findings from recent studies. A thorough literature search using electronic databases such as Scopus, PubMed and Google Scholar was conducted. Published articles between 2014 and 2025 were included. The search strategy employed combinations of terms including “women”, “reproductive age group”, “dietary diversity”, “diet quality”, “24-h dietary recall”, “urban informal settlement”, and “rural area”. Both observational studies and intervention trials that used a 24-h recall method for taking dietary history and studies reporting Minimum dietary diversity for women (MDDW) or dietary diversity score of women (WDDS) were considered for the review. Almost all the studies had reported inadequate dietary diversity in urban informal settlement and rural area except studies conducted at Nouakchott Mauritania (71.7%). The diets in urban informal settlement and rural area are often dominated by grains and tubers, pulses, milk and milk products, other vegetables with lower consumption of green leafy vegetables, eggs and vitamin A rich fruits and vegetables. Present scoping review highlights the persistent inadequacy of dietary diversity among WRA in both urban and rural areas.

## Introduction

Women of reproductive age (15–49 years) are particularly vulnerable for micronutrient deficiencies because of their higher micronutrient needs. Micronutrient deficiencies constitute a widespread nutrition challenge faced by women living in resource-poor settings. The consequences of which affect not only the health and survival of women but also their children, mainly through intrauterine growth retardation (1). In India according to National Family Health Survey (NFHS-5), approximately 57% of WRA were anemic (2). It was observed that the intake of micronutrient-rich foods was below 20% of the Recommended Daily Intake, and consumption of fruits and green leafy vegetables was significantly below the suggested level of 100 gm & 400 gm, respectively (3). In 2016 the United Nations initiated the “Decade of Action on nutrition” to intensify efforts to reduce malnutrition in all its forms with emphasis on integrated policy and program. But suboptimal micronutrient status remains a challenge in WRA group (4). The government of India has launched Poshan Abhiyan 2.0 to address the problem of malnutrition and to promote nutrition awareness and healthy eating habits for sustainable health (5). For reproductive age group women, diverse diets are to be prioritized as it ensures micronutrient adequacy (3). A less diverse diet is considered to be of lower nutritional quality and is associated with increased risk for cardiovascular disease and diabetes and increased mortality in women (6). Consuming a diversified diet over a given time period, commonly within last 24 h that includes food from different food groups is widely recommended as effective strategy to address micronutrient inadequacies (7). Diverse diet reflects availability, accessibility and utilization of food which are the three pillars of food security. The Food and Agriculture Organization (FAO) suggested minimum dietary diversity for women (MDD-W) as a validated nutrition sensitive indicator for assessing dietary diversity (8). In India urbanization has created distinct nutritional environments, with an estimated 65.5 million residing in urban informal settlement and 833 million in rural areas (9). These populations face barriers in achieving adequate dietary diversity like poverty and gender bias and incomplete knowledge about the diet quality (10). This review was conducted with the objective to map the evidence on dietary diversity among women of reproductive age (15–49 years) across rural and urban settings. This study provides a comparative rural urban analysis of dietary diversity among women of reproductive age addressing a key gap in the existing literature that mainly focuses either on urban or rural settings. It contributes to public health research by identifying disparities in access to diverse and affordable foods among rural and urban populations.

## Materials and methods

This is the scoping review addressing the research question—What is the evidence on dietary diversity among women of reproductive age (15–49 years) across rural and urban settings?

Present review was done using following steps – (1). Identifying the research question (2). Identifying relevant studies from the electronic databases and reference lists (3). Study selection was done by defining inclusion and exclusion criteria based on the research question (4). Charting the data of selected studies. (5). Collating, summarizing and reporting the results. Results of the review are reported using Preferred Reporting Items for Systematic Reviews and Meta-Analyses extension for scoping reviews guidelines (PRISMA-ScR).

Search strategy: Two reviewers (First author and second author) conducted the review. A Comprehensive literature search of relevant article was conducted from databases such as PubMed, Scopus and google scholar. Articles between January 2014 and December 2025 were included in the literature search. Search terms were in English. The database search terms included were “women”, “reproductive age group”, “dietary diversity”, “diet quality”, “24-hour dietary recall”, “urban informal settlement”, and “rural area”. Boolean operators And, OR were used to separate key words. Reference list of included articles was also searched for related studies. Observational studies and randomized controlled trials, were searched. Studies conducted in various settings including urban informal settlement and rural areas were included.

Eligibility criteria to select relevant studies on dietary diversity among women of reproductive age group was used – Inclusion criteria – (1). Articles reporting evidence on dietary diversity among women of reproductive age group (15–49 years) from rural and urban informal settlement area (2). Articles published in English language between January 2014 and December 2025 (3). Studies that used a 24-h recall method for taking dietary history.

Exclusion criteria – Articles reporting evidence on dietary diversity among pregnant and lactating women.

The selection process of studies included in the review has been demonstrated in Figure 1.

**Figure 1:**
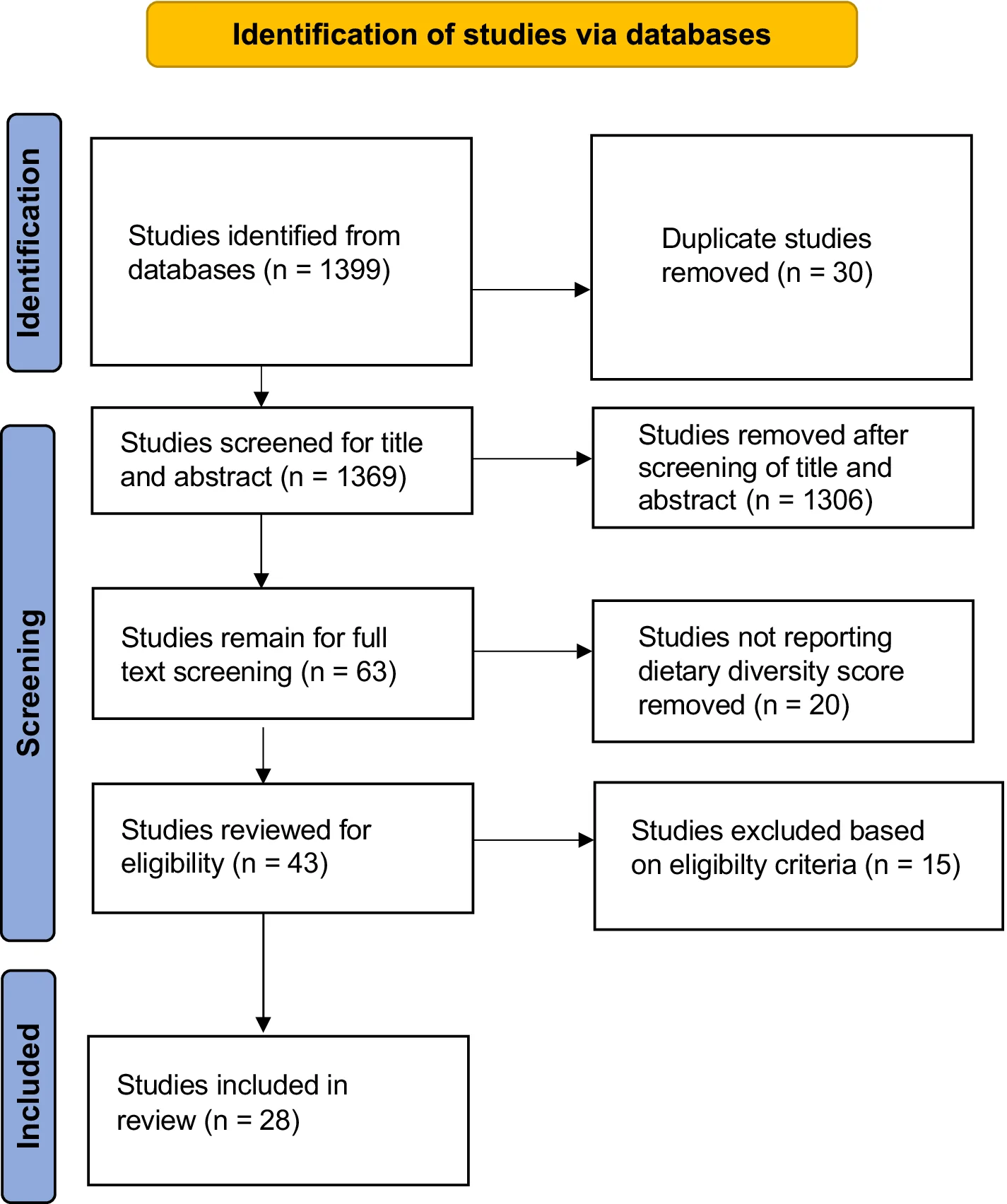
PRISMA flow diagram.

Based on the eligibility criteria, first one reviewer (SM) screened the titles from the databases. All eligible articles following title screening were exported into the Zotero library. Following title screening two reviewers (SM and JG) conducted screening of abstracts and full screening. Discrepancies in responses by the reviewers were resolved through discussion between the reviewers until consensus was reached. Data extraction sheet was prepared to extract data from selected studies. Data extraction included following information on author name, year of publication, study design, study area (Rural /Urban), sample size and main findings from each study. Two reviewers analyzed the data and result report was prepared.

In the studies included in this review the MDD-W score was calculated depending on the consumption of foods from 10 recommended food groups: (1). Grains, white roots and tubers; (2). Nuts and seeds; (3). Pulses; (4). Meat, poultry and fish; (5). Milk and milk products; (6). Green leafy vegetables; (7). Eggs; (8). Other vegetables; (9). Other vitamin A-rich vegetables and fruits; and (10). Other fruits. Women who consumed foods from five or more food groups in the previous 24 h were considered as having adequate dietary diversity (9).

## Results

### Burden of inadequate dietary diversity

The proportion of WRA achieving minimum dietary diversity varies significantly in urban and rural area. It was observed that in rural Ethiopia only 26.8% WRA, 27% WRA in rural Mali, in rural Rajasthan 23.5% and about 20% WRA from rural Burkina Faso achieved minimum dietary diversity, i.e., Consumption of ≥5 food groups (11–14). In Rural Ethiopia (Jeldu district) almost 81.9% had consumed at least five food groups (15). In rural area of Mandya district Karnataka 50.6% of WRA had a dietary diversity score of 5 and above (16). In rural Odisha India, almost 44% WRA had achieved MDD (6). In Urban and Peri- urban areas of Hyderabad, India, peri urban women had a mean dietary diversity score of 4 (17). In rural areas of Nashik district in Maharashtra 68.8% women had achieved minimum dietary diversity (18). In marginalized areas of Sri Lanka about 63%of WRA did not achieve MDD-W. Significant differences were found between urban and rural MDD-W (*p* < 0.05) (19). Among female residential students in Bangladesh, the mean dietary diversity score was 4.84 ± 1.02 (20). On St Martin’s Island in Bangladesh, the mean DDS was 4.25 and only 40.3% met MDD-W (3). In an informal settlement of Kolkata, India the proportion of women attaining MDD was 46.2% (10). In urban Patna Bihar, India almost 52.7% WRA had MDD less than 5 (21). The findings consistently reported that substantial proportion of WRA in resource poor settings do not meet the minimum dietary diversity score indicating micronutrient deficiency.

### Patterns of food intake

In both rural and urban setting diet were not diversified. WRA consumed predominantly starchy staples like grains, roots and tubers, pulses and other vegetables. Animal sourced foods like meat, poultry and fish, dark green leafy vegetables, eggs, nuts/seeds and fruits were least consumed (7, 10–12, 14, 20, 22). In rural Bihar improvement in diet quality is primarily because of consumption of fruit, dairy and vegetable groups but consumption of meat, poultry and eggs was low (23). In rural Mali, women with adequate dietary diversity had consumed animal source foods and /or vitamin A rich vegetables and fruits more frequently (12). In rural Tanzania it was reported high intake of grains/tubers, pulses/legumes and other vegetables. Meat /poultry/fish and dark green leafy vegetables were moderately consumed while eggs dairy and fruits were low (24). In urban areas of Sri Lanka WRA consumed meat, poultry, fish, dairy and pulses more often (>50%) while rural women consumed pulses, nuts/seeds, other vegetables (approximately 90%) and Vit A rich food (>60%) (20). In Ghana, women mostly consumed grains, tubers, roots (97.9%) and flesh meat (82.2%) Dairy (58.1%) dark green leafy vegetables (55.6%) and other fruits (63%) were moderately consumed (25).

### Factors influencing minimum dietary diversity (MDD-W)

In urban and rural settings dietary diversity among WRA has been influenced by various socio- demographic determinants.

*Educational status*: Higher educational status is significantly associated with improved dietary diversity among WRA (3, 11, 17, 26, 27).

### Decision making power and empowerment of women

Women’s participation in household decision making plays a vital role. Women with freedom to take decision in the family had more dietary diversity than other women (3, 15, 25).

### Food insecurity

Food insecurity significantly determines dietary diversity. Women living in the food insecure households were significantly less likely to achieve the Minimum dietary diversity (MDD-W) compared with those from food secure households (12, 28).

### Marital status

Marital status negatively impacts dietary diversity among WRA. Married women of reproductive age have a lower diet diversity as compared to single/never married women. (17, 21).

### Consumption of food groups

Starchy staples and other vegetables were the most commonly consumed food groups among participants, whereas intake of milk and milk products, animal source food, nuts and seeds and vitamin A -rich fruits and vegetables were limited. All participants reported consumption of grains (3, 7, 21). When grains or other vegetables-based staple foods dominate and diets lack fruits, green leafy vegetables and animal-source foods, then risk for micronutrient deficiencies is high which impacts women’s health (13).

### Dietary gap

Women had a slightly lower dietary diversity, consuming 0.1–0.5 fewer food groups than household members. The food groups responsible for this dietary gap are non-staples like, meat/fish/poultry, vitamin A-rich fruits and vegetables, and milk products (29).

### Cultural and religious practices

Cultural beliefs and religious fasting in Ethiopia adversely affect dietary diversity. Gender based discrimination, i.e., male members of the household eats first and females eat left over food and cultural food taboos influenced women’s consumption of animal sourced foods, with restriction on particular cuts of chicken and lamb. About (76.7%) of WRAs followed food restriction as a part of religious fasting practice. Negatively affect WRA diversity (12). Urbanization leads to a shift toward junk food consumption, which are easily available and more convenient than traditional nutritious food, which contributes to a double burden of malnutrition i. e. obesity and micronutrient deficiencies.

### Food source and access

Ownership of livestock, access to household vegetables and women’s freedom to visit markets were associated with improved dietary diversity. Access to nutrition and health information through social media was mediated by women’s economic status, as those with higher income were better able to afford mobile or radio and consequently demonstrated better dietary diversity (15).

#### Intervention approaches for improving dietary diversity

Various nutrition intervention strategies had been implemented. Nutrition education strategy show improvement in dietary diversity. Nutrition-sensitive agriculture (NSA) interventions at rural Odisha had shown improvement in dietary diversity of women of reproductive age group (25). In a study conducted in four districts of Uttar Pradesh & Bihar it was observed that women of reproductive age group who grew nutri-gardens had 0.38 times higher odds of having adequate dietary diversity (≥5) than WRA who did not (5).

## Discussion

Present scoping review highlights considerable rural – urban differences in dietary diversity among women of reproductive age. It has been observed that across the studies included in the review, dietary diversity does not merely depend on food availability but influenced by multiple factors like socioeconomic status, access to the market, education, agricultural production and decision-making power of the women for purchasing food etc. Study conducted in West Bengal has reported more mean diversity score in rural area compared to urban area (30). Easy availability and low price of the different food items in rural area compared to urban might have reflected as more dietary diversity and high individual dietary diversity score (IDDS) compared to urban area. In urban settings wide variety of food is available through markets but affordability remains a critical barrier to purchase them. Evidence from urban Burkino Faso demonstrates that higher household expenditure significantly improves likelihood of achieving minimum dietary diversity among women of reproductive age group (1). In urban Sri Lanka, households often purchase cheap, unhealthy and low-nutrient foods like oily fried snacks because women perceived that preparing food at home is more expensive than just buying food (19). In rural settings in Odisha better diet diversity scores observed because of improved socioeconomic status among women belonging to scheduled caste (6). Similarly in rural Bangladesh it was observed that increase in wealth significantly associated with increase in dietary diversity scores among women (26). Market access further differentiates urban and rural context. Women from the urban setting benefit from proximity to markets with diverse food options. Study in urban Burkina Faso revealed that choosing the place of purchase based on large choice rather than proximity showed a positive association with the dietary diversity (1). Contrary women in rural and geographically isolated areas such as those in island Bangladesh, experience limited access to low intake of sea foods, dairy products, nuts and seeds, and vitamin A-rich fruits and vegetables (3). These findings suggest that market access plays an important role in improving dietary diversity. While rural households are often engaged in agricultural production this does not translate into improved dietary diversity as agricultural production mainly centered around staple crops with less diversification. In some places such as St. Martin’s island in Bangladesh, dependence on imported foods further exacerbates inadequate dietary diversity (3). Urban population rarely engages in agriculture. However, women from peri urban Hyderabad are more involved in agricultural production as many men are rapidly leaving peri-urban areas leaving women with increased household and agricultural burden leading to lower dietary diversity as compared to their urban counterparts (17). Education of the women plays an important role in improving dietary diversity as education increases nutrition knowledge which helps in making healthy food choices. Educated women are more likely to participate in decision making like household food purchase, meal planning, allocating resources toward purchasing of nutritious food. In some studies, it was observed that unmarried women have better dietary diversity than married women. It might be because of the fact that unmarried women have few household responsibilities and better autonomy in food choices.

The findings from included studies provide consistent evidence linking inadequate dietary diversity with micronutrient deficiencies, although nature and strength of association vary across settings. In rural Odisha it was observed that women with adequate dietary diversity had 30% lower odds of mild anemia compared to those with lower dietary (6). Similarly, biochemical assessments in rural Pakistan revealed that women living in this community consume a less diverse diet and had high prevalence of zinc and iron deficiencies (4). Anthropometric Indicators and nutritional status: Participants with dietary diversity score of 5 and above have normal BMI range (21, 27). Studies from Karnataka and Patna suggested that women with higher dietary diversity are more likely to have normal body mass index (BMI) (4, 21, 31). This finding may reflect better access to food, improved socioeconomic status or overall dietary adequacy. However, evidence from southern Thailand demonstrated that adequate calorie intake does not necessarily translate into micronutrient sufficiency (7). This highlights the limitation of BMI as a sole indicator of nutritional status.

### Consistent patterns across settings

Analysis of the included studies in the present scoping review across Africa and southeast Asia reveals a consistent picture of nutritional vulnerability of women of reproductive age group as follows- Low prevalence of dietary diversity: Across nearly all settings a significant proportion of women have inadequate Minimum dietary diversity among women of reproductive age (MDD-W). Starchy staple dominance: Diets are characterized by mainly cereals, roots and tubers, often lacking milk products, meat, fish, eggs, vitamin A rich fruits and vegetables. Socioeconomic determinants: Education of women and wealth/ better economic status are the most consistent determinants of dietary diversity. Higher education leads to better nutritional knowledge. Also, better economic status improves food affordability which ultimately improves diet quality. Intra household gender bias: In South Asian contexts women often eat after men and eat leftover food contributing to inadequate dietary diversity. Agriculture production: In rural area agriculture production and ownership of livestock are significantly associated with adequate dietary diversity.

### Knowledge gaps

Seasonal variation in dietary diversity: Majority of the studies included in this review are cross sectional. Hence information on seasonal variation in the dietary diversity of women of reproductive age not available. Portion sizes and calorie intake: The MDD-W is a proxy indicator of micronutrient adequacy and does not measure portion sizes or caloric intake. There could be possibility that a woman may consume five or more than five food groups but still suffer from a caloric deficit if portion sizes are minimal.

### Strengths and limitations

This review provides a comparative rural–urban analysis of dietary diversity among women of reproductive age group, addressing a key gap in existing literature that mainly focuses on single setting studies. Also, it reveals how determinants of dietary diversity operate differently across different settings highlighting the role food environment, socioeconomic factors and cultural practices in shaping dietary patterns.

It is worth to note that there are some limitations in this review. Restriction to studies published between 2014 and 2025 may introduce selection bias. Lack of quality assessment of included studies limits the ability to judge internal validity and methodological rigor. There could be potential publication bias because of exclusion of studies in other languages than English. Majority of the studies rely on self-reported 24-h recall method. There could be recall bias or participants may over report healthy foods. Furthermore 24- h dietary intake history fails to capture day-to-day dietary variation.

## Conclusion

The studies included in this review demonstrate substantial heterogeneity in dietary diversity among women of reproductive age, driven by variations in geographic, socioeconomic, and cultural contexts. Evidence from regions across Africa, South Asia, and Southeast Asia highlights stark contrasts between urban and rural settings. There are challenges in achieving minimum dietary diversity among WRA in both rural area and urban informal settlement in developing countries. Though in urban areas there is availability of food variety but because of socio-economic constraints access to nutritious foods is limited which leads to inadequate dietary diversity in urban informal settlement. In contrast potential access to farm foods in rural areas, there are challenges like cultural factors, food insecurity and market access. Improvement in dietary diversity is important to increase micronutrients intake and overall nutritional status of WRA, which will positively impact maternal and child health outcomes. To promote the consumption of diverse diets multifaceted public health interventions should be implemented, including nutrition education, women empowerment, increase in decision making power of women and agricultural interventions that enhance the production and availability of nutrient-rich diverse crops.
