# Supplementary material for: Dietary diversity among women of reproductive age (15–49 yrs) across rural and urban settings: a scoping review
Source: Front Nutr. 2026 Oct 8;13:1893369. doi: 10.3389/fnut.2026.1893369 (PMC13688113; doi:10.3389/fnut.2026.1893369)
Supplement: Supplementary file 1 [file Data_sheet_1.pdf]

Preferred Reporting Items for Systematic reviews and Meta-Analyses extension for Scoping Reviews (PRISMA-ScR) Checklist

SECTION	ITEM	PRISMA-ScR CHECKLIST ITEM	REPORTED ON PAGE #
**TITLE**			
Title	1	Dietary diversity among women of reproductive age (15-49 yrs) across rural and urban settings: a scoping review	Title
**ABSTRACT**			
Structured summary	2	Dietary diversity (DD) reflects micronutrient adequacy and diet quality. Inadequate dietary diversity has adverse effects on health of the women and increases the risk of complications during pregnancy and childbirth due to deficiencies of micronutrients. This scoping review aims to synthesize the current understanding of dietary diversity among WRA in urban and rural settings, highlighting key findings from recent studies. A thorough literature search using electronic databases such as Scopus, PubMed and Google Scholar was conducted. Published articles between 2014 and 2025 were included. The search strategy employed combinations of terms including 'women' 'reproductive age group', 'dietary diversity', 'diet quality', '24-hour dietary recall', 'urban informal settlement' and 'rural area'. Both observational studies and intervention trials that used a 24-hour recall method for taking dietary history and studies reporting Minimum dietary diversity for women (MDDW) or dietary diversity score of women (WDDS)were considered for the review. Almost all the studies had reported inadequate dietary diversity in urban informal settlement and rural area except studies conducted at Nouakchott Mauritania (71.7%). The diets in urban informal settlement and rural area are often dominated by grains and tubers, pulses, milk &milk products, other vegetables with lower consumption of green leafy vegetables, eggs and vitamin A rich fruits and vegetables. Present scoping review highlights the persistent inadequacy of dietary diversity among WRA in both urban and rural areas	Abstract-structured summary
**INTRODUCTION**			
Rationale	3	This study provides a comparative rural urban analysis of dietary diversity among women of reproductive age addressing a key gap in the existing literature that mainly focuses either on urban or rural settings. It contributes to public health research by identifying disparities in access to diverse and affordable foods across rural and urban populations.	Introduction - Rationale
Objectives	4	To map the evidence on dietary diversity among women of reproductive age (15-49 years) across rural and urban settings to guide future research.	Introduction - Objectives
**METHODS**			
Protocol and registration	5	Study protocol is published and available on https://www.ijcmph.com/index.php/ijcmph/article/view/13956<br>Clinical trial number: CTRI/2025/02/080693(Dated 17/02/2025	protocol registration available on the given link

Eligibility criteria	6	Inclusion criteria – 1. Articles reporting evidence on dietary diversity among women of reproductive age group (15-49 years) from rural and urban informal settlement area 2. Articles published in English language between January 2014 - December 2025<br>3. Studies that used a 24-hour recall method for taking dietary history<br>Exclusion criteria – Articles reporting evidence on dietary diversity among pregnant and lactating women.	Methods – Eligibility criteria
Information sources*	7	. A Comprehensive literature search of relevant article from was conducted from databases such as PubMed, Scopus and google scholar. Date of recent accessed article- 3rd Feb 2026	Methods - Search strategy
Search	8	Articles between January 2014 to December 2025 were included in the literature search. Search terms were in English. The database search terms included were 'women' 'reproductive age group', 'dietary diversity', 'diet quality', '24-hour dietary recall', 'urban informal settlement' and 'rural area'. Boolean operators And, OR were used to separate key words. Reference list of included articles was also searched for related studies. Randomized controlled trials and observational studies were searched. Studies conducted in various settings including urban areas, peri- urban areas, urban informal settlement and rural areas were included.	Methods - Search strategy,
Selection of sources of evidence†	9	Based on the eligibility criteria, first one reviewer (SM) screened the titles from the databases. All eligible articles following title screening were exported into the Zotero library. Following title screening two reviewers (SM and JG) conducted screening of abstracts and full screening. Discrepancies in responses by the reviewers were resolved through discussion between the reviewers until consensus was reached. Data extraction sheet was prepared to extract data from selected studies. Data extraction included following information on author name, year of publication, study design, study area (Rural /Urban), sample size and main findings from each study. Two reviewers analyzed the data and result report was prepared.	Methods - Selection process
Data charting process‡	10	Data charting was done independently by two reviewers. Data extraction sheet was prepared to extract data from selected studies. Data extraction included following information on author name, year of publication, study design, study area (Rural /Urban), sample size and main findings from each study	Methods – Data selection Process
Data items	11	Women of reproductive age: Women between 15-49 years of age<br>MDD-W: Minimum dietary diversity for women<br>Adequate dietary diversity: Women who consume 5 or more than 5 food groups in last 24 hours considered having adequate dietary diversity.<br><br>24 – hour recall method: Structured assessment used to evaluate an individual's food and beverage intake over the past 24 hours<br>Urban informal settlement area: residential area characterized by substandard living conditions. These	Methods – Data items

		conditions typically include overcrowding, dilapidated housing, poor sanitation, inadequate infrastructure, and lack of basic amenities.<br>Rural area: A geographic area located outside towns and cities	
Critical appraisal of individual sources of evidence§	12	Not done	Not applicable
Synthesis of results	13	Based on the eligibility criteria, first one reviewer (SM) screened the titles from the databases. All eligible articles following title screening were exported into the Zotero library. Following title screening two reviewers (SM and JG) conducted screening of abstracts and full screening. Discrepancies in responses by the reviewers were resolved through discussion between the reviewers until consensus was reached. Data extraction sheet was prepared to extract data from selected studies. Data extraction included following information on author name, year of publication, study design, study area (Rural /Urban), sample size and main findings from each study. Two reviewers analyzed the data and result report was prepared.	Methods – Synthesis of results

SECTION	ITEM	PRISMA-ScR CHECKLIST ITEM	REPORTED ON PAGE #
**RESULTS**			
Selection of sources of evidence	14	Total 1399 articles identified from databases using the MESH terms. Out of these 30 articles were duplicate and hence removed. Remaining 1369 articles were screened for title and abstract. After this screening 1306 articles were removed. Hence 63 articles remained for full text screening. Out of these 63 articles 20 articles were removed as they were not reporting dietary diversity score. Remaining 43 articles were reviewed for eligibility. About 15 articles did not fit into the eligibility criteria, hence excluded. So total 28 studies included in this scoping review	Fig. 1 PRSMA flow diagram (Identification of studies via databases)
Characteristics of sources of evidence	15	Separate data extraction sheet is attached of the included studies.	Data sheet 2
Critical appraisal within sources of evidence	16	Not done	Not applicable
Results of individual sources of evidence	17	Separate data extraction sheet is attached of the included studies.	Data sheet 2
Synthesis of results	18	Almost all the studies had reported inadequate dietary diversity in urban informal settlement and rural area except studies conducted at Nouakchott Mauritania. The diets in urban informal settlement and rural area are often dominated by grains and tubers, pulses, milk &milk products, other vegetables with lower consumption of green leafy vegetables, eggs and vitamin A rich fruits and vegetables. Education, decision making power of the women are important determinants of improving dietary diversity.	Results – Synthesis of results
**DISCUSSION**			
Summary of evidence	19	Present scoping review highlights considerable rural – urban differences in dietary diversity among women of reproductive age. It has been observed that across the studies included in the review, dietary diversity does not merely depend on food availability but influenced by multiple factors like socioeconomic status, access to the market, education, agricultural production and decision-making power of the women for purchasing food etc. Women from the urban setting benefit from proximity to markets with diverse food options. Education of the women and decision-making power in the house, agricultural production plays an important role in improving dietary diversity	Discussion – Summary of evidence
Limitations	20	Restriction to studies published between 2014 to 2025 may introduce selection bias. Lack of quality assessment of included studies limits the ability to judge internal validity and methodological rigor. There could be potential publication bias because of exclusion of studies in other languages than English. Majority of the studies rely on self-reported 24-hour recall method. There could be recall bias or participants may over report healthy foods. Furthermore 24- hour dietary intake history fails to capture day-to-day dietary variation.	Discussion - Limitations

Conclusions	21	The studies included in this review demonstrate substantial heterogeneity in dietary diversity among women of reproductive age, driven by variations in geographic, socioeconomic, and cultural contexts. Evidence from regions across Africa, South Asia, and Southeast Asia highlights stark contrasts between urban and rural settings.There are challenges in achieving minimum dietary diversity among WRA in both rural area and urban informal settlement in developing countries. Though in urban areas there is availability of food variety but because of socio-economic constraints access to nutritious foods is limited which leads to inadequate dietary diversity in urban informal settlement. In contrast potential access to farm foods in rural areas, there are challenges like cultural factors, food insecurity and market access. Improvement in dietary diversity is important to increase micronutrients intake and overall nutritional status of WRA, which will positively impact maternal and child health outcomes. To promote the consumption of diverse diets multifaceted public health interventions should be implemented, including nutrition education, women empowerment, increase in decision making power of women and agricultural interventions that enhance the production and availability of nutrient-rich diverse crops.	Discussion-Conclusions
**FUNDING**			
Funding	22	No funding is available for conducting this scoping review	Funding

JBI = Joanna Briggs Institute; PRISMA-ScR = Preferred Reporting Items for Systematic reviews and Meta-Analyses extension for Scoping Reviews.

* Where *sources of evidence* (see second footnote) are compiled from, such as bibliographic databases, social media platforms, and Web sites.

† A more inclusive/heterogeneous term used to account for the different types of evidence or data sources (e.g., quantitative and/or qualitative research, expert opinion, and policy documents) that may be eligible in a scoping review as opposed to only studies. This is not to be confused with *information sources* (see first footnote).

‡ The frameworks by Arksey and O'Malley (6) and Levac and colleagues (7) and the JBI guidance (4, 5) refer to the process of data extraction in a scoping review as data charting.

§ The process of systematically examining research evidence to assess its validity, results, and relevance before using it to inform a decision. This term is used for items 12 and 19 instead of "risk of bias" (which is more applicable to systematic reviews of interventions) to include and acknowledge the various sources of evidence that may be used in a scoping review (e.g., quantitative and/or qualitative research, expert opinion, and policy document).

From: Tricco AC, Lillie E, Zarin W, O'Brien KK, Colquhoun H, Levac D, et al. PRISMA Extension for Scoping Reviews (PRISMAScR): Checklist and Explanation. Ann Intern Med. 2018;169:467–473. doi: 10.7326/M18-0850.
