# Supplementary material for: Dietary diversity among women of reproductive age (15–49 yrs) across rural and urban settings: a scoping review
Source: Front Nutr. 2026 Oct 8;13:1893369. doi: 10.3389/fnut.2026.1893369 (PMC13688113; doi:10.3389/fnut.2026.1893369)
Supplement: Supplementary file 2 [file Data_sheet_2.pdf]

Summary sheet of research articles included in the scoping review

Sr No.	Author(s)/ Year	Study design	Study area (Rural /Urban)	Sample size	Main findings
1	Custodio et al /2020 Apr DOI:10.1111/mcn.12897	Cross sectional	Urban Burkina Faso.	12 754 WRA	MDD-W in urban Burkina Faso was 31%, The most frequently consumed food groups were 'all starchy', 'vitamin A rich dark green leafy vegetables' and 'Other vegetables'
2	Islam MH et al /2023 Mar doi: 10.1186/s40795-023-00715-y	Acommunity-based cross-sectional study	St. Martin's island in Bangladesh	201 WRA	About 40.3% of the participants met the MDD-W cut-off. Educational level, and decision-making role of women were significantly related to their dietary diversity.
3	Brazier AKM/ 2020 Nov doi:10.3390/nu12113407.	Cross sectional	Rural community living in the brick kilns close to Peshawar, Pakistan	47 WRA	The mean (±SD) food group diversity score was 4 ± 1 with between 26% and 41% of participants achieving an MDD-W of 5. Women consume a diet that has a low diversity and zinc deficiency is prevalent
4	Priyanshu Rastogi et al /2023 DOI: https://dx.doi.org/10.18203/2394-6040.ijcmph20233105	Post-test-only comparison with a control group	one block in each of the two districts of Uttar Pradesh (Kaushambi and Varanasi) and Bihar (Jamui and Khagaria). The study sites were included as they were a part of the ongoing intervention by MAMTA	Total 804 women and adolescent girls (15-19 years) in the intervention and control groups	Women who grew Nutri-gardens had 0.38 times higher odds of having higher dietary diversity (≥5) than women who did not. Similarly, women who are educated have higher odds of having a higher dietary diversity compared to illiterates.
5	Jin Y et al /2022 May-Jun	Clustered randomized	Rural Odisha, India	980 non-pregnant	Forty-four percent of women were classified as

		controlled trial		women aged 15-49 years from Odisha, India	having a diverse diet (MDD-W ≥5). Women with higher education level, belonging to a scheduled caste (vs. tribe), and higher body mass index had higher odds of a diversified diet (p < .05 for all) Diet diversity was inversely associated with prevalence of mild anaemia.
6	Puwanant M et al /2022 doi:10.1186/s40795-022-00619-3.	Community-based cross-sectional study	Southern Thailand	120 healthy, young WRA	The most common foods consumed were rice, followed by meat, eggs, fruits, and vegetables. Fewer than 40% of the participants consumed beans, dairy products, vitamin A-rich fruits and vegetables, dark green vegetables, and pulses. The average MDD-W score was 5
7	Manika Pal, Bobby Paul, Aparajita Dasgupta/2017 10.5455/ijmsph.2017.0513114062017	A community-based, cross-sectional study	Slum of Kolkata	182 WRA	“Proportion of WRA” attaining “MDD” was found to be 46.2% only. There was significant association between dietary diversity and small family size, upper socioeconomic status AOR, higher educational level, and occupation other than homemaker
8	Alamirew SK, Lemke S, Stadlmayr B, Freyer B/(2023) doi: 10.3390/nu15153369	A community-based, cross-sectional study	Amhara region of Ethiopia	421 WRA	About 26.8% of the women consumed five or more food groups per day. Women’s education, age, perception of nutritious diet,

					and frequency of consumption of animal-sourced foods, vegetables, and fruit were significantly associated with MDD-W.
9	Adubra L, Savy M, Fortin S, Kameli Y/(2019) doi: 10.1093/cdn/nzz002.	A community-based, cross-sectional study	Rural area of Mali	5046 WRA	Only 27% of women reached the MDD-W. These women consumed animal source foods and/or vitamin A-rich vegetables and fruits. Women from extremely food insecure households were less likely to reach the MDD-W
10	Divya Raghuvanshi and Renu Mogra/2020/ DOI: 10.15740/HAS/ AJHS/15.1/110-113.	A community-based, cross-sectional study	5 villages of Udaipur district of Rajasthan state	200 WRA from 5 villages of Udaipur district. From each village 40 families	Only 23.5 per cent with Mean±SD 6.46±0.58 WRA had consumed more than five food groups. Consumption of cereals and other vegetables were the most commonly consumed food items consumption of meat and fish was negligible.
11	Loty Diop et al/2021	A community-based, cross-sectional study	Rural Burkina Faso is a low-income food-deficit country located in West Africa	1008 Non pregnant non lactating women	This study showed that FGSs based on either the MDD-W guidelines were positively associated with the women in rural Burkina Faso
12	Gudisa Merga et al /2022 https://doi.org/10.1371/ journal. pone.0279223	A community-based cross-sectional study	Jeldu District, West Shoa Zone, Oromia Region, Ethiopia	634 WRA	The proportions of women who consumed greater than or equal to five food groups were 81.9%. women who have a radio and women's decision-making power to purchase food for household and having own mobile

					phone were statistically associated with food dietary diversity.
13	Shashikantha SK, Sheethal MP/2016/ DOI: http://dx.doi.org/10.18203/2394-6040.ijcmph20160644	A cross-sectional study	Mandya district Karnataka, India	77 WRA	Majority of them had dietary diversity score (DDS) above 5 . Subjects with normal BMI range had better odds of having DDS of 5 and above
14	Kiran Suryasai Marla et al /2023 https://doi.org/10.1186/s40795-023-00692-2	A cross-sectional study	Selected urban (1108 individuals) and peri-urban (808 individuals) locations of Hyderabad, India.	n = 1816: 660 adult males, 662 adult females, 205 adolescent males, and 289 adolescent females.	Peri-urban adult women consume a less diverse diet compared to their urban counterparts. Marital status, type of household card owned, and the highest degree of education are statistically significant correlators of an individual's dietary diversity.
15	Doke PP, Chutke AP, Palkar SH/ (2025) https://doi.org/10.47203/IJCH.2025.v37i03.005	A cross-sectional descriptive study	Nashik district Maharashtra, India	7,794 women.	The mean dietary diversity score of the women was 4.73 (SD=0.7), 68.8% had minimum dietary diversity. Starchy food consumption was 100%, and fruit and vegetable consumption were minimal.
16	Permani C. Weerasekara, Chandana R. Withanachch, G. A. S. Ginigaddara, Angelika Ploeger/2020/ doi:10.3390/foods9111659	Cross-sectional study	Urban slums and remote rural areas in Sri Lanka	400 WRA	About 63% of reproductive age women did not meet MDD-W and food security. significant positive correlation between MDD-W and area, monthly income, educational

					level, food source, BMI level and health status.
17	Sultana M, Hasan T, Shaheen N/2019/ doi: 10.12944/CRNFSJ.7.1.24	Cross-sectional study	Bangladesh	180 WRA	Starchy staples were the main food group consumed by the respondents
18	Puja, Rafat Perveen, Sneh Smriti, Surendra Prasad Singh/2024	Cross-sectional study	Urban Patna, Bihar, India	150 WRA	Participants with BMI range ≥21.5 Kg/mt2 had better odds of having MDD score of 5 and above.
19	Issa MY, Diagana Y, Khalid ELK et al. /2024	Cross-sectional study	Urban area of Nouakchott, Mauritania	240 WRA	The mean of dietary diversity was 5.48 and 71.7% of WRA had an acceptable minimum dietary diversity. The consumption of legumes, eggs and other fruits was low.
20	Kalyani Raghunathan et al /2023/ https://doi.org/10.1016/j.worlddev.2023.106267	Cluster-randomized controlled trial. The JEEViKA-intervention	Three blocks of Saharsa district of Bihar		A 30% increase in the proportion of women achieving minimum dietary diversity but had no impact on women's body mass index.
21	Bellows AL, Canavan CR, Blakstad MM et al /(2020);41(1)doi: 10.1177/0379572119892405	Baseline survey of Cluster randomized controlled trial (RCT)	Rural Tanzania	1006 WRA	Ten percent of women in this study reported consuming an average of 5 or more food groups per day. Starchy staples and flesh foods were the most commonly consumed food groups. Women in households that owned livestock, participated in cash crop production, grew pulses, and grew other vegetables were significantly more likely to have high dietary diversity.

22	Amugsi, D.A., Lartey, A., Kimani-Murage et al/ (2016). https://doi.org/10.1186/s41043-016-0053-1	Data from the Ghana Demographic and Health Surveys (GDHS)	Ghana	2262 WRA	The odds of achieving higher DD were higher among women who had a say in deciding household purchases, Women who had more than primary education were 1.6 times more likely to achieve higher DD.
23	Helen Harris-Fry et al /2015 DOI 10.1186/s41043-015-0022-0	A community-based cross-sectional study	Rural Bangladesh	2,809 WRA	Women reported consumption of an average of 3.8 (range 1, 9) food groups over one day. Women's freedom to shop alone was limited;. Sixty three percent of respondents reported that they (alone or with their husband) were the main decision-makers for purchasing daily necessities such as food.
24	Smita Kumari Panda et al /2019 DOI: https://doi.org/10.32553/ijmbs.v3i6.283	Community based cross sectional study	Urban field practice area of VIMSAR,Burla	150 WRA	"Proportion of WRA" attaining MDD was 47.30%. All of study subjects consumed cereals. Least number of people consumed food of nuts and seeds group.
25	Weigel MM, Armijos RX, Racines M, Cevallos W/2016 doi: 10.1155/2016/8149459	Community based cross sectional study	Quito, Ecuado	794 WRA	Household Food Insecurity prevalence was high (81%) and associated with several undernutrition indicators suggesting micronutrient malnutrition in the urban Quito participants
26	Gupta S, Sunder N, Pingali PL/2020/doi: 10.1177/0379572120943780	Community based cross sectional study	Urban India	3600	Women consistently consume 0.1 to 0.5 fewer food

					groups relative to other household members. The food groups driving this dietary gap are nonstaples like Vitamin A-rich fruits and vegetables, meat/fish/poultry, and dairy
27	Archan Mukherjee, Sourabh Paul, Indranil Saha/2018/ DOI: 10.4103/mjdrdypu.mjdrdypu_15_18	Community based cross sectional study	Rural and urban area , West Bengal India	216	Mean diversity score was more in rural compared to urban area. rural population had consumed significantly more of fish, meat, egg, and green leafy vegetables compared to urban.
28	Apprey C, Addae HY, Boateng G./2025/ doi: 10.1111/mcn.13757	Community based cross sectional study	Urban Ghana	407 WRA	About 67.3% WRA were with adequate dietary diversity . WRA consuming monotonous carbohydrate-based diet
